# Objectivity applied to embodied subjects in health care and social security medicine: definition of a comprehensive concept of cognitive objectivity and criteria for its application

**DOI:** 10.1186/s12910-018-0254-9

**Published:** 2018-03-02

**Authors:** Hans Magnus Solli, António Barbosa da Silva

**Affiliations:** 1 Research Unit, Division of Mental Health and Addiction, Vestfold Hospital Trust, PO Box 2168, NO-3103 Tønsberg, Norway; 2 Ansgar University College and Theological Seminary, Fredrik Fransonsvei 4, NO-4635 Kristiansand, Norway

**Keywords:** Cognitive objectivity, Clinical assessments, Criteria of objectivity, Epistemology, Interpretation, Intersubjectivity, Ontology, Phenomenology, Professional expertise, Work disability

## Abstract

**Background:**

The article defines a comprehensive concept of cognitive objectivity (CCCO) applied to embodied subjects in health care. The aims of this study were: (1) to specify some necessary conditions for the definition of a CCCO that will allow objective descriptions and assessments in health care, (2) to formulate criteria for application of such a CCCO, and (3) to investigate the usefulness of the criteria in work disability assessments in medical certificates from health care provided for social security purposes.

**Methods:**

The study design was based on a philosophical conceptual analysis of objectivity and subjectivity, the phenomenological notions ‘embodied subject’, ‘life-world’, ‘phenomenological object’ and ‘empathy’, and an interpretation of certificates as texts. The study material consisted of 18 disability assessments from a total collection of 86 medical certificates provided for social security purposes, written in a Norwegian hospital-based mental health clinic.

**Results:**

Four necessary conditions identified for defining a CCCO were: (A) acknowledging the patient’s social context and life-world, (B) perceiving patients as cognitive objects providing a variety of meaningful data (clinical, psychometric, and behavioural data – i.e. activities and actions, meaningful expressions and self-reflection), (C) interpreting data in context, and (D) using general epistemological principles. The criteria corresponding to these conditions were: (a) describing the patient’s social context and recognizing the patient’s perspective, (b) taking into consideration a variety of quantitative and qualitative data drawn from the clinician’s perceptions of the patient as embodied subject, (c) being aware of the need to interpret the data in context, and (d) applying epistemological principles (professional expertise, dialogical intersubjectivity, impartiality, accuracy and correctness). Genuine communication is presupposed. These criteria were tested in the work disability assessments of medical certificates. The criteria were useful for understanding both how objectivity fails during work disability assessments and how it can be improved in the writing of certificates.

**Conclusion:**

The article specifies four necessary conditions for the definition of a CCCO in health care and social security medicine and the corresponding criteria for its application. Analysis of the objectivity of work disability assessments in medical certificates for social security confirmed the usefulness of the criteria.

## Background

Objectivity is a contested concept in health care and social security medicine. ‘Objective finding’ is the traditional criterion of objectivity, based on the biomedical model of disease. It is also the officially sanctioned criterion of objectivity in social security [1]. However, in most descriptions of mental illnesses and conditions involving so-called ‘medically unexplained symptoms’, no objective findings are present. In such cases, an issue arises about criteria of objectivity that could substitute for objective findings. Moreover, in medical practice, it seems that objectivity is affected by personal or social interests: human subjectivity lurks everywhere. In this article, we define an epistemological concept of objectivity that takes the pervasiveness of human subjectivity into account, and specify the practical criteria for its application. In an analysis of the objectivity of work disability assessments in medical certificates for social security, we explore whether the criteria are useful and fruitful or not.

Our analysis of the concept of objectivity takes a pre-scientific starting point: not from objective findings of what is in the body alone, but from the perception of a patient or claimant of social security benefits as a whole person. This presupposes a holistic concept of the human being as constituted by body, soul and spirit, forming an integrated whole [2]. Accordingly, a common-sense understanding of objectivity is the point of departure for our analysis. In human social life certain data, facts or states of affairs exist that are there for everyone to perceive, examine or discuss together with others. This assumes that both perceptions and arguments have a public character. People can confirm, or disconfirm, the validity of one another’s perceptions, arguments or statements. In this sense, a basic common-sense understanding of objectivity means in principle *validity for everyone*.

Philosophers have defined the concepts of objectivity and subjectivity in fruitful ways [3–7]. It is relevant for this study to distinguish between three senses of objectivity and subjectivity: ontological, epistemological and ethical. We shall use definitions of ontological and epistemological (or epistemic) objectivity by John Searle as our starting point.

Searle has defined the concept of ontological objectivity (hereafter *O-objectivity*) as follows:

[T]he ontological sense [of objectivity] refers to the status of the mode of existence of types of entities in the world. Mountains and glaciers have an *objective mode of existence* because their mode of existence does not depend on being experienced by a subject ([5], p. 44).

‘Objectivity’ as an ontological term refers to a mode of existence that is independent of experience by a subject. For our purpose, we maintain that the ontological sense of objectivity, as traditionally used in the natural sciences, refers to material entities. It also represents a common-sense view of reality according to which what is there for everyone to perceive and agree upon is material reality.

We now come to the definition of O-objectivity as applied to medicine. In the natural sciences – of which biomedicine is a part – O-objectivity was earlier often regarded as the only concept of objectivity. To say that an objective finding is O-objective is to say primarily that what is found is something that exists ‘outside’ experience [1]. An ‘objective finding’ of this kind is pathological ‘reality’ as seen and felt by a surgeon or pathologist. The concept of the objective finding so defined presupposes that neither the patient’s nor the physician’s consciousness (subjectivity) affects the content and rigour of a medical assessment.

We wish to emphasize that in certain biological systems such as the human being, subjective reality is fundamental [5, 8]. Searle writes that the concept of ontological subjectivity (*O-subjectivity*) refers to a mode of existence of a kind that exists ‘only as experienced by some human or animal subject’. Examples are ‘pains, tickles, and itches, as well as thoughts and feelings’ ([5], p. 44). Other examples of O-subjective reality are: illness, pain, anxiety, depression, well-being, quality and meaning of life. Thus, subjective reality exists, but only as entirely dependent on consciousness.^1^ Subjective realities have a quality of felt experience, or awareness. To describe them correctly one needs to take a first-person viewpoint, i.e. the viewpoint of the experiencing subject. In the present context, this means the view of the patient or claimant. It has a first-person ontology, as Searle maintains ([5], p. 52).

Aspects of human communication also have a first-person viewpoint, sometimes expressing the experience of being not only an ‘I’, but also a ‘we’[9], p. 43f). At other times, O-subjectivity in human communication embodies an experience of the other person as a ‘you’, as Martin Buber has clarified in relation to genuine dialogue [10]. This is a second-person viewpoint. Both the first- and the second-person viewpoints express the specific character of human consciousness, which is an ontologically subjective phenomenon.

When discussing epistemology, following Nicholas Rescher [3], we use the term cognitive objectivity (*C-objectivity*) as a synonym of epistemological objectivity. And we use cognitive subjectivity *(C-subjectivity*) as a synonym of epistemological subjectivity.

With the concept of C-objectivity, ‘a statement is considered objective if it can be known to be true or false independently of the feelings, attitudes, and prejudices of people’ ([5], p. 44). With regard to science, Searle maintains that ‘[s]cience is indeed epistemically objective in the sense that scientists try to discover truths that are independent of anyone’s feelings, attitudes, or prejudices’ [5], p. 45). He adds: ‘So the fact that consciousness has a subjective mode of existence does not prevent us from having an objective science of consciousness’ ([5], p. 45). It is possible to have a science of psychology, for example. Description both of ontologically objective entities and subjective phenomena can be C-objective.

Like other sciences, medicine is a C-objective science. The American Medical Association’s *Guides to the Evaluation of Permanent Impairment* display a C-objective definition of objective findings, typically defining ‘objective finding’ in terms of quantitative measurements (‘Objective Test Results’):

[…] for example, X-rays, computed tomography (CT), magnetic imaging (MRI), laboratory tests, electrocardiography (ECG), electromyography (EMG) – with specific findings that confirm or validate the diagnosis and/or indicate severity of the particular condition. As tests they are the most objective source of data available […]. ([11], p. 15)

The test apparatus described here, is based on C-objectivity. The particular abnormal findings are to be read and interpreted by a specialist with qualified knowledge.

‘Objective finding’ is also defined cognitively as ‘a sign that can be seen, heard, felt or measured’ ([12], p. 1316). This definition broadens the scope of what can be found objectively, not only by a test battery, but also, for example, by what the physician observes from the patient’s general appearance, e.g. distress and changed posture, walk, and motor activity, and also the specific findings of the professional examination of an embodied human being [13]. Another doctor would have observed approximately the same abnormalities, i.e. the observations can be C-objective. It should be noted that C-objectivity occurs in degrees, i.e. it is possible to be more or less objective, since the assessment of objectivity may vary with the clinician or expert who undertakes it.

We believe that C-objectivity is appropriate to a medical understanding of objective findings. A ‘finding’ is never like an exact photography of reality. It is the result of an interpretation of signs of abnormalities or pathologies of the living human organism, an interpretation that is always carried out in the terms of professional expertise. In medicine, objective findings must be seen primarily as C-objective.

To sum up so far: We have indicated three kinds of human domains found in health care: (i) the human body as a physical entity existing independently of our perception of it, (ii) the psyche or mind (depression, angst, joy, despair, meaninglessness, etc.), which has an O-subjective existence, (iii) the embodied human being, whose ontology we come back to below. All of these human domains can be studied in a C-objective way; and in all these domains, cognitive data implies, as we conceive it, the existence of something outside the data itself as the ontological reference of the data.

One recognized definition of C-objectivity is *intersubjectivity,* not least in science today ([14], p. 23). ‘Intersubjectivity’ implies that a statement is ‘established as true, probable or acceptable by procedures that in principle can be followed by everyone’ (our translation) ([15], p. 350). This presupposes intersubjective communicability. Defined in this way, the meaning content of ‘intersubjectivity’ is close to that of ‘confirmability’ as a methodological requirement. As will be shown, intersubjectivity is important in the analysis below.

We have already introduced the first- and second-person viewpoints. We now introduce the third-person viewpoint. The third-person viewpoint is the observer’s point of view. It is the point of view of the scientist, appropriate for an object of the natural sciences. An important inference from Searle’s concepts is that, because consciousness has a first-person ontology, it “cannot be reduced, or eliminated in favour of, phenomena with a third-person ontology” ([5], p. 52). In other words: We can have a science of the third-person aspect of consciousness, but the aspect of first-person ontology, of subjective experience, cannot be reduced to third-person ontology. We conclude that C-objectivity has to take into consideration the reality of three irreducible viewpoints: first-person, second-person and third-person. All three viewpoints are necessary to describe the living, communicating human being – as constituted by the dimensions body, soul and spirit – living in interaction with environments [2].

Searle writes that a statement is *C-subjective* ‘if its truth depends essentially on the attitudes and feelings of observers’ ([5], p. 44). More examples of such attitudes and emotions are prejudices, passions, biases, loyalties, conformities, allegiances and unsupported opinions ([3], p. 5) In daily life, people in general have a variety of such first-person standpoints, which they may recognize as important for themselves. In professional scientific contexts, however, C-subjectivity should be avoided as far as possible. What is important is to be as aware of one’s prejudices and preconceptions as one can.

Concerning the essential features of C-objectivity, David Bell writes that the distinction between objectivity and subjectivity in epistemology

[…] serves to distinguish two grades of cognitive achievement. In this sense, only such things as judgments, beliefs, theories, concepts and perceptions can significantly be said to be objective or subjective. Here objectivity can be construed as a property of the contents of mental acts and states. ([4], p. 310)

We agree with Bell in supporting Immanuel Kant’s insight that the above-mentioned property entails ‘presumptive universality’ which means that: for a judgment to be C-objective it must, at least, possess a content that ‘may be presupposed to be valid for all men’ ([4], p. 310). Hence, C-objectivity is a cognitive property of mental acts and statements that in principle make them valid for all rational humans, at least in the same historical cultural context. Practically, this is done by stating valid reasons supporting judgements, beliefs, assessments, theories, concepts or perceptions as objective. In a nutshell: C-objectivity claims general validity, *based on reasons* [1].

To secure, as far as possible, general validity in concrete situations, epistemological principles are necessary in applying C-objectivity. One meta-principle is that rationality should be exercised with an appropriate goal in mind ([3], p. 9). Applied to the present study, this means that certificates should be written with a stated commission in mind [1]. The application of this methodological rule is presupposed in the following analyses. Other principles for the application of C-objectivity are *impartiality*, a*ccuracy* and *correctness* [1]. We shall come back to the use of epistemological principles below.

Objectivity has also an ethical sense, linked to the concept of impartiality, which is commonly also understood as a principle or criterion of justice as fairness. Often viewed as synonymous with fair-mindedness, impartiality holds 'that decisions should be based on objective criteria rather than on the basis of bias, prejudice, or preferring to benefit one person over another for improper reasons’ [16]. To objectively balance conflicting interests, duties and goods in social collaboration requires impartiality.

We have now defined the concepts of objectivity and subjectivity as a necessary condition for defining the cognitive concept of objectivity that takes into account human subjectivity. This concept has to avoid two types of ontology that are still widespread: (a) Cartesian substance dualism (‘everything is either matter or mind’, i.e. matter and mind are two separate and independent realities), and (b) a reductionist monistic materialism (‘everything is only matter’, i.e. subjectivity does not really exist) [2, 17]. We believe that both O-objectivity and O-subjectivity are necessary conditions for understanding living beings such as the human one, but they should not be regarded as separate and independent of each other. This is a fundamental aspect of a holistic and multidimensional view of the human being [2]. A cognitive concept of objectivity which takes into account this view of the human being in health care and social security is what we term *a comprehensive concept of cognitive objectivity* (CCCO). Below we shall define the CCCO and explain the criteria for its application in health care.

## Methods

### Aim

The aim of the study was three-fold. The first aim was to specify some necessary conditions for the definition of a CCCO, which enable objective descriptions and assessments even of subjective phenomena in health care. The second was to formulate some necessary criteria for the application of CCCO. The third was to investigate the application of these criteria in a collection of work disability assessments in medical certificates for social security purposes written in a mental health care context.

### Design

The study was based on a theoretical design consisting of two interacting parts. The first part used conceptual analysis to specify some necessary conditions for the definition of a CCCO and the resulting criteria for application of the CCCO in health care and social security medicine. The analysis was carried out by having in mind a variety of assumed objective work ability or disability assessments in a collection of texts consisting of medical certificates. The second part used the defined criteria to make reasonable interpretations of the objectivity of work disability assessments in medical certificates issued for social security purposes, regarded as texts. By ‘reasonable interpretation’ we mean an interpretation allowed by the rules of grammar, semantics and logic and the context of the text. Hence, the interpretation was carried out, *inter alia*, from a hermeneutical point of view, which emphasizes that meaning arises within contexts and that the interpreter of a text is influenced, among other things, by his or her pre-understanding and cultural context [18]. Details regarding how the analysis was carried out are found in our earlier article [19].

### Setting

The social and cultural setting of this study is a social welfare system of the Nordic type in Norway. There is a close relationship between the Norwegian health care system and the Norwegian Labour and Welfare Administration (NLWA). Hence, clinicians have two roles to handle, one as a practitioner treating the patient and another as an expert writing certificates to third parties on demand. The claimants are long-term patients at two units in the Division of Mental Health and Addiction at the Vestfold Hospital Trust in Southern Norway (‘the clinic’). Certificates written by both psychiatrists and psychology specialists (‘experts’) working at the clinic constitute the data for this study.

### Material

Certificates from the clinic, commissioned by the local office of the NLWA, were collected over 3 years between 1 January 2007 and 31 December 2009. Questions concerning the patient’s medical details and possible educational or vocational activities were answered. Details of the procedure by which dis-identified copies of the certificates were produced and received running numbers have been described elsewhere [19]. In all, the material consisted of 86 medical certificates issued for social security purposes in respect of 66 claimants (43 women and 23 men) between the ages of 19 and 64 years (median age 40 years). They were written by 12 psychiatrists and 12 psychology specialists. The material represents 28% of the claimants and 65% of the eligible experts. For the present article, the 18 disability assessments from this material were studied. In the quotations from the assessments given below, the certificate being quoted is identified by its running number.

## Necessary conditions for the definition of a comprehensive concept of cognitive objectivity (CCCO)

### The embodied subject

We must first reflect on how the embodied human being should be envisaged. We believe the concept of *lived experience* provides a fruitful way of approaching a CCCO for practical use in health care. An appropriate method of describing lived experiences is *phenomenology*, which is also an area of philosophical study and of understanding of actual human experience (German: *Erlebnis*), especially ‘the ways things present themselves to us in and through such experience’ ([20], p. 2). Our analysis in this article shows that aspects of the lived experience not only of the patient or claimant, but also of the clinician, have to be taken into account.

Maurice Merleau-Ponty represents an approach within phenomenology that combines philosophical phenomenology with empirical sciences. We follow this approach in dealing with the human being.^2^

We have drawn on Merleau-Ponty’s concept of the *embodied subject* in fleshing out the conceptual structure below [17, 21, 22]. The human body is both biological organism and lived experience. Biological organisms are not isolated things, as the science of ecology shows. Neither is lived experience something that occurs in a mind/body shut in on itself. A basic bodily experience is that ‘my body is a movement toward the world and […] the world is my body’s support’ ([21], p. 366). The embodied subject has to be understood as life that stretches out towards and is supported by its surroundings. Hence, human bodies should be basically understood as interacting with one another and with their surroundings. Merleau-Ponty writes that ‘we must rediscover the social world […], not as an object or sum of objects, but as the permanent field or dimension of existence […]’ ([21], p. 379). In the present article, the concept of embodied subject expresses the concrete living human being, where material embodiment, bodily experience of being in the world, and social, cultural and social environments are regarded as dynamically linked ([17, 23, 24], pp. 159–175). In the analysis below we have employed the following concepts from the phenomenological tradition: *life-world*, *phenomenological object* and *empathy*. In stating four necessary conditions for the definition of CCCO in health care, we have combined these concepts with the concepts of O-objectivity, O-subjectivity, C-objectivity and C-subjectivity as defined above under ‘Background’.

### First condition: Acknowledgement of the patient’s social context and life-world

The WHO has acknowledged the importance of social context in its development of the *International Classification of Functioning, Disability and Health, ICF* (hereafter ICF) [25]). The ICF attempts to integrate the medical model with a social model ([25], p. 20). The manual describes human functioning in terms of body integrity, individual activities or actions in environments and participation in social life [25]. In his study of medical practice, Eric J. Cassell emphasizes the patient’s functioning using the terms of the ICF [26]. The ICF has taken important steps towards recognizing the social context of human functioning.

There are, however, basic problems with the ICF. Its medical model of interpretation is still based on reductionist monistic materialism [2], and hence it provides only the third-person viewpoint. Important concepts such as intention or goal – which are integral elements of an action – are not included among the components of the ICF [2]. Rehabilitation doctors have struggled for recognition of the subjective dimension of functioning and disability [27]. The phenomenological notion of *life-world* (German: *Lebenswelt)* can fill out the shortcomings of the ICF in relation to subjective experience.

Edmund Husserl described the life-world as the concrete and immediate world of everyday experience [28]. This world is pre-scientific and is experienced even before ‘the split between physical and psychical‘ ([28], p.189). ‘Life-world is an all-embracing term that includes the “surrounding world” (*Umwelt*), both that of nature and culture, including humans and their societies (“the world of culture”), things, animals, our overall environment’ ([28], p. 190). We believe that ‘life-world’ is the appropriate overarching ontological term in health care for the unity of the human, social world *as it is experienced* by the embodied person. The life-world encompasses first-, second-, and third-person viewpoints as already defined above under ‘Background’. It is important to note that, because a person’s life-world includes first- and second-person viewpoints, there will always be limits to how far the life-world can be described objectively. Merleau-Ponty writes that ‘the social exists silently and as a solicitation’ even before we ‘come to know it or when we judge it’ ([21], p. 379). ‘Life-world’ is now an established term in psychiatry and psychology [29, 30].

### Second condition: The patient perceived as a cognitive object providing a variety of data

The second condition is based on an understanding that the patient, as an embodied subject, appears as a living *cognitive object* to an observer – in this case a clinician – in a variety of ways when the latter is in authentic communication with the patient. To explain this point, we need first the phenomenological notion of the *phenomenological object.* It is a mental object or ideal entity, and not physical, as is the usual sense of the word ‘object’. Karl Jaspers defines the concept of phenomenological, i.e. intentional object (German: *Gegenstand*) as follows:

We give the name ‘object’ in its widest sense to anything which confronts us; anything which we look at, apprehend, think about or recognize with our inner eye or with our sense-organs. In short anything to which we give our inner attention, whether it be real or unreal, concrete or abstract, dim or distinct. Objects exist for us in the form of perceptions or ideas ([31], p. 60).

We shall follow this definition, but add to it cognitive aspects of emotions [8]. The quotation above is an example of a fundamental philosophical insight that conscious states are intentional: they are about, or refer to, intentional objects [5]. They are called ‘intentional’ because they are often directed by consciousness towards something (the intended object), which could be, for example, other people, the environment, numbers, facts, states of affairs, signs, data or plans for the future. They can also be about the subject’s own ego, psyche or mind.

According to phenomenology, perceptions, ideas and emotions, as described above, typically have cognitive contents, namely their intentional objects. Phenomenology combines properties of the object ‘outside mind’ with the experience of ‘inside mind’ into a unified cognitive act. In this act, as human beings we are related both to objects in the external world, to other human beings and to our own experience, and in this way meaning is formed. ‘[T]he meaning of things, in a sense, exists neither “inside” our minds nor in the world itself, but in the space between us and the world’ ([22], p. 34). Applied to healthcare, the data from the patient acquire meaning in the interaction between the patient and the clinician. Such meaningful data are here termed ‘cognitive objects’. We introduce the concept of cognitive object (Jaspers’ phenomenological object) in this study to expand the application of C-objectivity to the human being as embodied subject.

To explicate further the cognitive object in the interpersonal context, we need the phenomenological concept of *empathy*. Empathy is the ability to understand and share the feelings of another. Phenomenologically, empathy is intentionality directed at the experiences of the other person. Understanding comes into being by perceiving the other person in context. This understanding is both emotional and cognitive. Imagining the other person in his/her life arenas is also important [32, 33]. Empathy is recognized as a fundamental phenomenon in human interaction and communication [34].

Intentional objects are perceivable and communicable intersubjectively. Phenomenology explains this basically in terms of the concept of empathy, which ‘allows us to experience behaviour as expressive of mind. [Empathy] allows us to access the feelings, desires, and beliefs of others in their expressive behaviour. Our experience and understanding of others is [however] fallible’ ([35], p. 155). Empathy is a means to intersubjective understanding. We shall come back to the concept of dialogic intersubjectivity below.

Merleau-Ponty’s view of intentionality – as pre-predicate unity of the experienced world and life – helps us to become aware that not all aspects of a problematic relationship between a person with ill health and the work market (a work disability) can be accessed as cognitive objects, i.e. as available to our knowledge ([21], p. lxxxii); or, as Searle underlines, not everything that a human being experiences can be accessed as cognitive objects by others (i.e. from the third-person viewpoint). Examples are ‘[u]ndirected feelings of well-being or anxiety are not intentional’ ([36], p. 327). In Searle’s terms this means that some of patients’ or claimants’ undirected feelings, including their well-being and anxiety, cannot be accessed as intentional or cognitive objects. If well-being as an undirected feeling cannot be accessed completely as a cognitive object, its opposite, permanent ill health, also cannot be fully described as an object for other persons. This is interesting in our context, because a common understanding of work disability is that it is often complex and sometimes difficult to describe and explain in full. However, ill health can still be described as a narrative (see below).

We now describe the ways in which embodied subjects present themselves and provide data for clinicians, divided into clinical, psychometric and behavioural data, as follows.

### Data from clinical examinations

The concept of the embodied subject fully includes scientific data from the human organism and its illnesses and impairments. Descriptions of signs from clinical examinations in the different specialties of medicine and psychology are fundamental cognitive objects.

### Psychometric data

Psychometric data are obtained through psychological tests. Psychology is defined as ‘the study of the nature, function, and phenomena of behaviour and mental experience’ ([37], p. 619). Seen this way, we can say that psychometric data obtained by psychological tests belong to the third-person viewpoint, the point of view of the observer. However, evaluating a psychological test is a challenging cognitive activity. Important questions are the test’s theoretical orientation, practical issues, standardization norms, reliability and validity [38]. Nevertheless, reflectively carried through, psychometrics provides a way of obtaining meaningful data relating to the embodied subject.

### Behavioural data

In health care, behaviour can often be understood as reaching out towards fellow human beings in terms of what Jaspers calls ‘expressions’. He maintains that the ‘psyche and body are one for us in expression’ ([31], p. 225). We use Jaspers’ broad concept of behaviour to characterize objects or data relating to the embodied subject in terms of activities/actions, expressions and reflection. Behaviour has to be understood through empathy ([[31], pp. 251–97). Other philosophers also acknowledge that mental life expresses itself through the body. P. M. S. Hacker, writes that ‘behaviour is not only bare bodily movements, but smiles and scowls, a tender or angry voice, gestures of love or contempt, and what the person says and does’. Such behaviour ‘manifests the inner’ and runs counter to Cartesian substance dualism ([39], p. 45).

#### Activities and actions in environments

Jaspers describes the psychiatric patient as an active human being in terms of a variety of objective performances ([31], pp. 168–221). Since he wrote that in 1959, WHO has developed the ICF to describe human functioning [25]. Environmental facilitators and barriers are public phenomena. Abilities (and competences) are also phenomena that can be spoken about in a public or cognitively objective way.^3^

#### Meaningful expressions of mind/body relation

The concept of meaningful expression, which is publicly visible, comprises ‘meaningful objective phenomena’ (Jaspers) such as:

- Life in the individual’s ‘own personal world’, the place where the individual ‘by means of his attitudes, behaviour, actions […gives shape] to his environment and social relations‘ ([31], p. 251). Or, in other terms, the life-world of the individual so far as it can be perceived by another person.
- Postures, movements, gestures, facial expressions, gazes, and tones of voice ([31], pp. 253–74).
- A drive to express oneself in different ways: speech, written productions, drawing, art and handicraft, and individual outlooks of the world ([31], pp. 287–97).

#### Self-reflection

Reason as the capacity for reflection is fundamental in the human world. Reflecting on one’s own goals or intentions is a part of being a rational being. This is because an important quality of the person is that he or she is an *agent*, that is, an *acting being* [2]. A person’s intention or goal is part of the world of reason. In clinical work, too, there are opportunities for the clinician and patient or claimant to reflect together ([31], p. 274). A patient’s/claimant’s intention or goal is therefore a cognitive object that the clinician and the patient/claimant can reason and deliberate about.

To sum up: The second condition for the definition of the CCCO enables clinicians to perceive the patient as a living, cognitive object providing a variety of data, both quantitative and qualitative.

### Third condition: The interpretation of data in context

To make sense, the myriad of meaningful data about a patient or claimant have to be interpreted by the clinician. They need to be interpreted in light of the social context, the purpose of the assessment, and clinical knowledge. This third condition calls for specific attention to the ways in which the perceived data are interpreted by a clinician. The clinician will use his or her knowledge and experience to make sense of the interpretation in the current context. Clinical interpretation has two aspects: one is in some way to describe the patient’s lived life, the other to make a professional assessment of themes of that life. The first can be described as a narrative, the second as a theoretical interpretation [40]. The latter uses scientific models. Daniela Bailer-Jones defines a scientific model as ‘an interpretive description of a phenomenon that facilitates access to that phenomenon’ ([41], p. 1). This definition is useful for the use of practical models in health care, too.^4^ We study work (dis)ability models below.

A basic aspect of interpretation is the circular relationship between the whole and its parts. In our context, this means that the data can only be understood when aspects of the patient’s life-world as a whole – daily routine, different activities, health condition, social relationships, cultural setting and so on – are taken into consideration [26]. Similarly, the patient’s life-world taken as a whole can only be understood in relation to the data on each of these aspects. When working out this interpretative, i.e., hermeneutic circularity, the clinician will ask the patient/claimant questions, comparing the information given against experiences from his/her own life-world and experience of being an embodied subject. Sometimes it is relevant to check for coherence and consistency among the data provided as components of a life narrative.

In this clinical activity, the ethical sense of objectivity comes to the fore. “[M]edical professionals have a particular obligation to create situations where it is possible for patients to present themselves as subjects with integrity and legitimate opinions” [42]. When writing certificates, questions about the credibility of a claimant’s presentation of data will sometimes come to the mind of the expert [1]. Sometimes, degrees of symptom magnification or occasional malingering have to be considered [43]. This requires a reasonable interpretation of the collected data.

### Fourth condition: The use of epistemological principles

The first three conditions involve perceiving and assessing a patient/claimant in the *particular* relationship between the patient/claimant and the clinician. The fourth condition consists in the use of *general* epistemological principles for objectivity. Epistemological principles should be used to ensure the validity of interpretations, descriptions and judgements of what is perceived, understood and assessed as C-objective. Well-known epistemological principles for application of C-objectivity are the following [1]:

#### Intersubjectivity

C- objectivity was defined in terms of intersubjectivity under ‘Background’ above. Applied to clinical assessments, by ‘intersubjective validity’ we mean ‘what is the case/evident or true according to current professional expertise’. This means that an account should be built upon available facts or data, and that it should be supported by arguments [44]. (Germanic terms are *saklighet* [Norwegian] and *Sachlichkeit* [German]). What is clinically described or assessed should be intersubjectively communicable and testable by other professionals in the same or similar contexts.

In psychotherapy, the practice of intersubjectivity is specified as a kind of interpersonal exchange that, following Buber (see above under ‘Background’), in this article is called *dialogic intersubjectivity* [10, 45]. In certificates, for example, the concept of dialogic intersubjectivity is found in use when the expert refers to important life-world events that the expert and patient/claimant have talked about together (a cognitive object as described in the second condition for the CCCO above). An interpretation of the patient’s problem situation is constructed by the patient and therapist together. This interpretation is then written out in a certificate as an account of some relevant and important aspects of the patient’s/claimant’s life and work history. The intersubjective communication is primarily between two subjects, but the account (in the certificate, in this example) is written in such a way that it can be understood and considered to be objectively valid not just by those two persons but by any competent reader.

#### Impartiality

An account should not be twisted by the omission of some information that the writer (e.g. of a certificate) ought to understand is important for the receiver and for the purpose of the certificate. The descriptions should be factual and sober and not biased or tendentious [1].

#### Accuracy and correctness

The information should be copious enough that the receiver can imagine the claimant’s situation for him/herself, thus allowing misconceptions about the real situation to be avoided [1].

A greater degree of objectivity is ensured by using these principles. When they are not used, C-subjectivity can result.

### From defining conditions to criteria for their application

The CCCO has been defined above in terms of four necessary conditions. These conditions can now be expressed as the following criteria for the application of a CCCO in health care and social security medicine:

#### First criterion

To take into consideration the patient’s/claimant’s social context and, when appropriate, also life-world (lived experience). At the least, important aspects of the patient’s social context (e.g. close relatives) should be considered. First- and second-person perspectives should be recognized.

#### Second criterion

To take into consideration a variety of quantitative and qualitative data from the clinician’s empathic perceptions of the patient as a cognitive object.

#### Third criterion

To be aware of the need to interpret the data in terms of both the patient’s/claimant’s lived experience and of a professional assessment.

#### Fourth criterion

To apply general epistemological principles to ensure objectivity in the concrete situation.

The use of all these criteria presupposes genuine communication between the expert and the patient/claimant.

To sum up: The patient/claimant should be seen as a whole human being, and listened to in his/her social context. When appropriate, relevant aspects of his/her life-world should be appraised. The clinician should recognize the patient as an embodied subject, not as a merely physical object, i.e. as his/her (clinician’s) own cognitive object, perceived through the use of empathy and imagination. The clinician should use his/her interpretive capacity to understand the variety of data from the patient/claimant, in the context of his/her clinical knowledge and experience and the purpose of the assessment. To ensure objectivity of assessments, it is important to use some generally recognized epistemological principles in the concrete situation.

### Application of the criteria of the CCCO in medical certificates for social security

The material on which this analysis is based consists of social security certificates written by psychiatrists and psychology specialists in their role as experts to determine claimants’ eligibility for social benefits. It is a requirement that such assessments should be objective. In Norway, the law prescribes that ‘[a]nyone who issues medical certificates, medical reports, etc., shall be careful, precise and objective’ ([46], §15). The government admits that claimants diagnosed with illness without objective findings, but with credible chronic disability, are also eligible for disability benefit. This has provided greater room for the use of professional discretion concerning the objectivity of assessments of work disability.

The certificates were written in a context where the claimant and the expert had met each other for at least one interview in the hospital setting. The expert had access to the patient’s earlier medical files, and in addition often knew aspects of the patient’s life from ongoing or earlier treatment spells. The certificates were all written in such a way that the reader understands that the expert has empathy with the claimant.

All the 18 certificates that constitute the material for this study concluded with ‘at least 50% work disability’, for a few years ahead or permanently. No disability is described in the texts in terms of objective findings. The main reason for this seems to be that no certificate contains a diagnosis from the group of organic mental disorders (ICD 10 diagnostic block F00-F09).

It should be noted that the expert assessing disability is not obliged to conclude with any specific quantification of the level of work disability. The specific level, 50% or higher, is decided by the NLWA on the basis of information about loss of income, and often also on reports from work ability training or work ability testing in various settings.

According to Norwegian law, a certificate from a health care expert should usually contain three parts: (a) background and relevant history, (b) a description of current data from clinical examination and interview, and (c) assessments and conclusions or recommendations [47]. In this article, we study (b) in terms of descriptions of the patient or claimant’s present functional disability in relation to work. We also study (c).

We do not study whether or not the requirements of the law – that appropriate treatment and attempts to return to work have been completed – are fulfilled in the *legal* sense. We have no information as to whether the claimants’ applications for disability benefit were granted or not.

### Work (dis)ability models as structuring devices for our interpretation

We have seen above that the use of professional practical models is important in fulfilling the third criterion of application of the CCCO – that concerning data interpretation. We therefore use work (dis)ability models to structure our interpretation of disability assessments. In our earlier study we found that the following three models of work (dis)ability are implicitly in use in the text collection as a whole [19]

- *The biomedical disability model (BDM):* This model ‘views disability as a problem of the person, directly caused by disease, trauma or other health condition’ ([25], p. 20). The basic question is whether a person’s disability is caused by a disease or not. A disability is described in only general and medical terms [19]. Earlier analyses have shown that the form used in work (dis)ability assessment is structured according to the BDM, with ‘objective finding’ as the fundamental criterion of objectivity. The basic sense of the concept seems to be O-objective [1]. It is not obligatory for experts to use this form.
- *The ability-based health model (AHM):* This model is based on action theory and on the holistic and relational definition of health by Lennart Nordenfelt [48]. Three components of the definition constitute what we have termed *health factors*. These are: (a) ability (or capacity), which in the ICF is a qualifier of the component activity [25]; (b) environment, described by the ICF in terms of barriers or facilitators; and (c) goal or intention – a factor not considered in the ICF [2, 19]. For an assessment to be qualified as using the AHM, the expert must describe the claimant holistically in a particular context, giving specific details about all three of the health factors specified above. The factors abilities and goals are examples of data belonging to the second criterion of a CCCO.
- *The mixed health model (MHM):* This model is intermediate between the BDM and the AHM. The BDM is most often used as a base, but with one, two or three of the above mentioned health factors added to the descriptions [19]. In some other descriptions, health factors are described without taking the BDM into account.

All three of the models of work (dis)ability are found in use in the collection of the 18 disability assessment texts analysed in this study. We investigated whether these disability assessments apply the four criteria of the CCCO, and if they do, in what way. Where the fourth criterion is concerned, we focus on professional expertise, dialogic intersubjectivity and accuracy. Here, we highlight our most striking interpretive findings.

### General causal assessments based on the BDM

In one group of 10 certificates, the BDM is used exclusively in three certificates; in the other seven, the BDM is used as the basic model, but the claimant is described by a few words in a social context, meaning that the model used is the MHM. Table 1 shows the reasoning process in one of the assessments based on BDM alone (cert.12). A greatly reduced ability to work is worded as ‘owing to prolonged depression’. Permanent, complete work disability status is recommended by the expert. The two other certificates based on BDM alone demonstrate the same reasoning process in the case of a middle-aged woman with 20 years of dependence on psychoactive substances. Functioning was described only in relation to symptoms or impairments in these three certificates.

**Table 1:** Some work disability assessments (translated from Norwegian). The claimant’s main diagnosis (ICD-10) is given

*Cert. 7:* ‘Development of depression, little ability to set limits and a great deal of stress in close relationships [have] eventually made her unable to function at work. It is known that it takes a very long time for a person to return to occupational functioning after developing such a state, and it is unlikely that she will be able to gain employment within the foreseeable future’ (elderly woman, F33).
*Cert. 12:* ‘Her ability to work is assessed as greatly reduced owing to prolonged depression. In addition, she is much preoccupied with pain and what this is doing to her. Her condition is assessed as chronic. She will, as she now appears, not be able to function in any job, nor in job training. She is assessed as disabled in the ordinary job market. Further treatment in psychiatric secondary care is not indicated’ (middle-aged woman, F33.1).
*Cert. 43:* Relationships with immediate family, especially a daughter with special needs, are described generally. There is no information as to whether the daughter’s situation has affected the claimant’s disability or not. The described activity limitations refer to interpersonal interactions: ‘Poor ability to impose herself, speak up about her own opinions, needs etc.’ Appropriate treatment is described as completed. No measures to improve her work ability are reported. She is judged to be permanently disabled, suffering from chronic ‘reduced perseverance, pain in her body after effort, has to rest for days afterwards’. The expert writes that ‘the causes of her physical complaints seem obscure, but underlying psychological condition and personal factors have at least been contributing factors’ (middle-aged woman, F41.8).
*Cert. 57:* ‘Assessment: X now wants to gain a disability pension. Based on what is described above, he believes that he will not be able to get into employment again. If he is required to do so, his anxiety level will increase significantly, with the risk of alcohol abuse and hence increased risk of suicide. He is now more satisfied with his life than for many years, he has control with respect to alcohol, he has leisure pursuits that he is satisfied with, and he feels that he gradually has learned to come to terms with life. He has made use of the therapy hours here at the clinic in a very satisfactory manner and has gained insight into how his life has been the way it has. [...]. The patient is now [an elderly man] who is seeking disability benefits. The undersigned supports him in this. For the patient, having to deal with a job with many obligations now will destroy the life he has today’ (elderly man, F60.6)
*Cert 73:* This is a claimant who is described as having been addicted to drugs since he was a teenager. He is currently on medication-assisted treatment. ‘The extensive and continuous drug addiction has resulted in major social-medical problems. During treatment it has emerged that he no longer believes that he can cope with work or work training. His image of himself is very poor [….]. I assess the patient as having had 100% reduction in work ability for many years, and I find it hard to envisage that this will change in the future’ (middle-aged man, F11.2).

Among the certificates that included a few words about the social context, cert. 43 (Table 1) assesses a claimant as work-disabled, but comments that the cause of the disability seems to be obscure. Cert. 73 (Table 1) gives a condensed summary of another claimant’s situation. The expert formulates a hard-hitting argument by grounding it in a description of poor functioning and negative self-image over the course of many years. The expert seems to be saying: Trust me, this claimant is permanently unable to work. There are two more certificates in this group that describe relationship stress as having significance for the work (dis)ability. One of these is cert. 7 (Table 1), which describes a claimant living with a husband suffering from chronic excessive alcohol consumption. Two common features of this group of certificates are the following:

- Only a third-person viewpoint is employed.
- The assessments are short and focused on the causal relationship between illness and work disability. Aspects of the claimant’s life situation are not included in the assessments, or only to a small degree.

These features are not surprising, as the BDM is strictly scientific and does not include social contexts other than labour services [19]. A basic deficiency in these descriptions is that the social context is not described well enough to explain the disability. These assessments do not fulfil the first criterion to take at least the patient’s social context into consideration.

Concerning the second criterion of the CCCO in this group of certificates, the assessments are based on standard clinical examinations. Neuropsychological examination was the only psychometric test used as part of the work disability assessment (*n* = 2). Behavioural data (belonging to the second criterion) are, however, not provided. The certificates do not distinguish between the claimants’ experience and the professional assessment. This is in accordance with the form used, which does not make this distinction. The third criterion is not fulfilled.

Concerning the fourth criterion of the CCCO, to apply general epistemological principles, we tested the application of the principle of professional expertise. When the sentences assessing the work disability are analysed, as they were in our study, the permanent disability that is claimed on the basis of causal reasoning alone is unconvincing. The descriptions lack some information that would demonstrate more clearly the reasoning from premises to a conclusion. The assessments based on the BDM fulfil the principle of professional expertise to a lesser degree than if social context had been described more closely. This point is demonstrated in one of the certificates when a broader social description in the medical history is taken into account (cert. 12, Table 1). This certificate states that the claimant’s suffering is caused by a range of disabilities: ‘limited knowledge of the Norwegian language, dyslexia, lack of school education, economic problems’ and also ‘lack of desire to recover’. It is likely that these mainly social disabilities were an important background to the final assessment. We can also say that the 10 assessments based on the BDM do not fulfil the principle of accuracy because they lack important information about the claimant’s social context.

### A social medical assessment based on the MHM

One MHM-based certificate (cert. 81, a middle-aged woman, diagnosis F60.7), using the BDM as the underlying model is, however, different from the others. It uses the NLWA form, but transcends it by describing a demanding social situation in the family in some detail, but still only from a third-person viewpoint. The husband is on permanent disability benefit and has his own problems, and two of their three children have special needs at school. The claimant is described in terms of symptoms, but also of activity limitations: ‘She has great interpersonal problems and also has problems of taking care of her own needs and those of her family’. She is assessed as having ‘such great personal and family difficulties that she appears unable to work’. The expert also argues that in reality she has not worked for 10 years. This certificate fulfils the first criterion of taking the social context into consideration. The description enables the reader to understand the claimant’s difficult social situation and to follow the reasoning process towards the conclusion of long-term work disability. The epistemological principles of professional expertise and accuracy are to some extent fulfilled.

### MHM disability assessments where the BDM is left out

The remaining certificates (*n* = 7) are structured as the experts themselves choose. One common feature of these certificates is that the assessment includes more aspects of the claimant’s life situation. The claimant’s subjectivity also comes to the fore.

Five of the seven certificates have been interpreted as applying the MHM, but without using the BDM as a base. These certificates are predominantly written in a third-person viewpoint, but they also approach the first-person viewpoint. The first-person viewpoint is expressed clearly in a certificate when what the claimant thinks, feels or experiences is quoted directly. None of the certificates in our study material does this. However, in these five certificates the experts state the claimant’s opinion and use this statement of the claimant’s first-person viewpoint in the argument in favour of work disability. This is thus a use of what we term the *close to the first-person viewpoint*. One expert writes about a claimant that he was fired from a job in which he had invested a lot of his strength and sense of responsibility: ‘When he was made redundant he collapsed, and he has realized that he cannot face entering into new arrangements to try out his work ability’ (cert. 35). Another expert writes: ‘To questions about his thoughts on employment, he says that he never will dare to meet others in an office or working place’ (cert. 16). The descriptions fulfil the first criterion of context and lived experience more than the previous certificates discussed.

The certificates distinguish between descriptions of the claimants’ opinions, etc., and the professional assessment. It is clear from the assessments that the experts have given some weight to claimants’ opinions. We emphasize that four of them are the first ones among the certificates studied to fulfil explicitly the third criterion**:** To be aware of the need to interpret the data in terms of both the patient’s experience and the professional assessment. The fifth, however, does not distinguish clearly between the claimant’s opinions and the expert’s own professional view of these opinions. It is therefore an interesting case. The certificate relates to a claimant who was unable to complete a work training programme at an appointed place because the requirement to attend 2 days a week created great anxiety in him. As can be seen in the assessment part of cert. 57 (Table 1), the claimant’s opinion has permeated the expert’s assessment. This is clear from the following statements: ‘He believes that he will not be able to get into employment again. If he is required to do so, his anxiety level will increase significantly, with the risk of alcohol abuse and hence increased risk of suicide.’ It looks as if the expert has taken the claimant’s opinions at face value. The data regarding the claimant’s opinion have entered into the expert’s assessment in a direct way. This assessment does not fulfil the third criterion.

### Narrative and dialogic intersubjectivity based on the AHM

The last two certificates briefly describe abilities, environments and goals, all in the particular context of the work-disabled claimant, i.e., the AHM is used. Due to lack of space, we shall analyse only the most detailed certificate here (cert. 44, a young woman, diagnosis F48.00). The text in it is introduced as follows: ‘Knowledge of the patient’s education, work experience and occupational training is taken as granted. Other information is here given fully, because it is considered significant in explaining her level of functioning to-day’. Information about the claimant’s work disability is given in terms of a life narrative.

Cassell has characterized a narrative in the health-care context, the following aspects of which are relevant to our study. A narrative should reveal ‘the chain of events that led to the present state’. It should explain both causative factors and the patient’s ‘purposes and goals’. The ‘meanings that the patient has attached to what has and is happening’ also belong to it, as do ‘the patient’s values. What the patient thinks is important’ ([49], p. 93). We analyse cert. 44 with these aspects in mind.

First, fundamental influences on the claimant’s situation in her childhood are described: an alcohol-abusing and violent father, and an unstable, chronic sick mother. She was sexually abused for 5 years in childhood and was bullied at school.

Second, the claimant’s moral standards and important actions are described. The expert writes that she managed to stop her incipient drug abuse and also tried to help others to stop their drug abuse. ‘She has always felt responsibility in the family and has been a prop and mainstay for everyone’. She now has a ‘secure family life’ with a husband and children. Central themes in the appointments with the expert have been her worries for her sick mother, her children, her own failing ability to work and social isolation. Her deserving efforts are emphasized.

Third, she has suffered and been treated for, among other things, asthma and chronic muscle pain. For as long as she can remember, she has struggled with mental problems and great burdens.

Fourth, at present she is in despair, because she feels she has no control and is at the mercy of her life situation. She feels guilty for not managing to give her children a better childhood.

Functionally, she is described as powerless and unable to mobilize strength. She is said to be unable to go outside with her children as she wants. She has handed over large parts of the housework to her husband. She manages to care for the family’s dog and has a close female friend whom she meets regularly. The narrative describes an ‘intertwinement of action and passion’ in human life ([50], p. 266).

The descriptions are written from a third-person viewpoint, especially in regard to the claimant’s childhood. As can be seen from the quotation above, the ‘close to the first-person viewpoint’ has also been used. We interpret this narrative also as written from a second-person viewpoint. The life history is based on a clinical dialogue that has been going on for some years. We regard this certificate as fulfilling the first criterion: a description in terms of the patient’s life-world.

The certificate demonstrates the kind of cognitive objects and data that belong to the second criterion: Important activities and actions (taking responsibility, helping others), struggling with failing ability to work and social isolation, reduced functioning and self-reflection. Because some behavioural data are described, we can say that the second criterion is fulfilled.

The two AHM-based certificates distinguish clearly between the narrative data expressed by the claimants and the experts’ assessments of these data. The third criterion is fulfilled.

In regard to the fourth criterion, we assess this narrative as fulfilling the epistemological principle of dialogic intersubjectivity. Cassell has an illuminating description of this kind of collaborative activity: ‘[I]t is true to call the doctor the historian while the patient is the storyteller’ ([49], p. 92). The narrative is a joint product between two collaborating subjects. It also fulfils the principle of accuracy. This is an example of what Cassell points out about narratives. They ‘include attitudes and valence – the emotional force – of the teller […]’ ([49], p. 92). It is also an example of how the ethical sense of objectivity comes to the fore. This sense is closely related to the virtue of justice conceived as fairness.

The assessment in cert. 44 interprets the claimant as a ‘traumatized and vulnerable young woman who seems to have stood upright in the family since she was a child’. The assessment explains why the claimant’s childhood traumas remain untreated. The assessment concludes that the claimant is long-termed disabled. The reasoning process from premises to conclusions fulfils the condition of professional expertise.

## Discussion

We have carried out an exploratory, interpretive study of a small set of texts originating in disability assessments in medical certificates produced for social security purposes. Four criteria of application of the comprehensive concept of cognitive objectivity (CCCO) have been tested.

We believe that the way in which certificates are written at the clinic that provided the study certificates is representative of that found in the Norwegian mental health care clinics [19]. The 18 certificates analysed should ensure typical ways of describing work disability in social security certificates. It is, however, likely that greater variation exists among work disability assessments than was observed in this study [19]. A limitation of this study is that certificates in which ‘objective findings’ were described were not included, and so the functions of this criterion of objectivity in relation to disability could not be studied.

Our findings suggest that it makes a significant difference whether the long-term disability of claimants with mental illness is assessed using the BDM, with ‘objective findings’ as an implicit criterion of objectivity, or using C-objective criteria. When the BDM is used, the work disability assessments tend to be short and focused on determining the causal relationship between work disability and illness. The social context is sparsely described, and the descriptions of the case lack sufficient accuracy. Both the factual grounds and the BDM as warrant are insufficient to support the conclusion that the claimant is permanently disabled. Using the BDM is inappropriate when there are no objective findings, and a relative lack of objectivity is found in such assessments. Objectivity improves when the BDM is supplied with social medical data.

In the two certificates where the AHM is used, the patient’s context has been extended somewhat. These certificates also describe the patient’s close to the first-person viewpoints and loss of abilities, in addition to the patient’s goal and reflections. The data are varied and relevant for a disability assessment. In these certificates, the experts use their own practical model as warrant for concluding that the claimant is permanently disabled. We do not know more about the practical models used than that the claimant is seen as an agent, in relevant context and having failing abilities. The objectivity of the assessments is improved. We do not know, however, the specific content of each expert’s warrant that made them conclude that the claimant is disabled.

In discussing Merleau-Ponty’s and Searle’s notions of intentionality above, we implied that not all aspects of a person’s work disability or ill health are accessible or available to our knowledge from the third-person viewpoint. Describing chains of important life events narratively in a text is a way of externalizing actions and experiences for both the patient and the clinician. ‘Once produced, the text becomes a matter for public interpretation’ ([29], p. 335). Writing a narrative seems to be a useful way of describing a complex work disability in an intersubjective way.^5^ However, it seems to be difficult to state the exact reasons why a patient or claimant is permanently work disabled.

So far as the concept of objective finding is concerned, ‘objective finding’ as defined primarily O-objectively is necessarily inappropriate in most medical conditions in health care today. However, if, for example, a claimant has advanced cancer, the O-objective pathological reality of the cancer underlines the severity of his/her condition. The claimant is obviously permanently disabled for work. On the other hand, we do not believe it is appropriate to designate all the C-objective descriptions and assessments that can be found by perceiving a claimant as a cognitive object as ‘objective findings’. We believe that the concept of C-objective finding should be restricted to the results of the clinical test apparatus *plus* the signs of abnormality or pathology that can be found by clinical examination of the embodied subject. We believe that the time has come to allow objective findings to find their important but limited place among the other criteria of the CCCO.

## Conclusions

The study has defined a CCCO for use in health care and social security medicine that ensures holistic thinking about human beings. Well-accepted definitions of ontological objectivity and subjectivity, and epistemological objectivity and subjectivity, provided the point of departure for the conceptual analysis undertaken here. It was found that the C-objectivity is appropriate to a medical understanding of objective findings. To expand the understanding of ontological subjectivity as related to material reality, the phenomenological notions of embodied subject, life-world, phenomenological object and empathy were included in the conceptual analysis. The CCCO was defined by four conditions. The criteria corresponding to these conditions for the practical use of the CCCO in health care are: (1) To take into consideration the patient’s social context and, when appropriate, also life-world (lived experience). The patient’s perspective should be recognized (2). To take into consideration a variety of quantitative and qualitative data from the clinician’s perceptions of the patient’s life and the patient’s test results. (3) To be aware of the need to interpret the data in context. (4) To apply general epistemological principles (professional expertise, dialogic intersubjectivity, impartiality, accuracy and correctness) in the concrete situation. The use of all the criteria presupposes a genuine communicative interaction. The concept of CCCO also comprises the ethical sense of objectivity, which takes into consideration respect for human vulnerability, dignity, individual identity, autonomy and integrity.

The four criteria were tested in an exploratory manner on the disability assessments contained in a collection of medical certificates written for social security purposes. The criteria were illuminating and useful in an analysis of what makes disability assessments for social security purposes more or less objective. The findings of our analysis suggest that the four criteria constitute a useful tool to aid an understanding of how objectivity in work disability assessments fails or can be improved or safeguarded. There is, however, a need to test the structure of the concept and the criteria in various arenas in health care where objectivity of clinical assessments is important.

## Abbreviations

AHM: Ability-based health model (a work (dis)ability model)
BDM: Biomedical disability model (another work (dis)ability model)
CCCO: Comprehensive concept of cognitive objectivity
Cert.: Certificate
C-objectivity: Cognitive objectivity (another term for epistemological objectivity)
C-subjectivity: Cognitive subjectivity (another term for epistemological subjectivity)
MHM: Mixed health model (a third work (dis)ability model)
NLWA: Norwegian Labour and Welfare Administration
O-objectivity: Ontological objectivity
O-subjectivity: Ontological subjectivity

## Footnotes

1 Consciousness has two different senses: first, consciousness in the sense of the human mind or ego, and, second, consciousness as awareness of something. The former is the subject or agent of all human perception, understanding, thinking and remembering, imagining, etc. The latter is a psychological state of the former in its perception, understanding, thinking, conceiving, etc.

2 Shaun Gallagher [51] and Evan Thompson [52] are two representatives of this approach in our time.

3 Jaspers has an interesting comment: ‘We can grasp and investigate only what has become an object to us. *Psychic life as such is not an object* [our italics]. It becomes an object to us through that which makes it perceptible in the world, the accompanying somatic phenomena, meaningful gestures, behaviour and actions. It is further manifested through communication in the form of speech. It says what it means and thinks and it produces works. These demonstrable phenomena present with the effects of the psyche.’ ([31] , p. 9)

4 We will not enter into a discussion of evidence-based medicine here, but remark that Peter C. Gøtzsche distinguishes in an illuminating way between four types of clinical thinking as the basis for clinical decisions in the context of evidence-based medicine. They are: (1) scientific theoretical, (2) scientific empirical (divided into uncontrolled and controlled experience), (3) empathic-hermeneutic humanistic and (4) ethical thinking [53]. A group of health scientists has recently called attention to the need to make explicit how interpretation is ‘involved when combining and applying the knowledge components’ in evidence-based medicine [54].

5 It should be noted that one should be careful with use of the term ‘objectification’ in interpersonal contexts. This is because ‘objectification’ often connotes the act of treating a person as a thing [55].
